# Automated tractography in patients with temporal lobe epilepsy using TRActs Constrained by UnderLying Anatomy (TRACULA)

**DOI:** 10.1016/j.nicl.2017.01.003

**Published:** 2017-01-05

**Authors:** Barbara A.K. Kreilkamp, Bernd Weber, Mark P. Richardson, Simon S. Keller

**Affiliations:** a Department of Molecular and Clinical Pharmacology, Institute of Translational Medicine, University of Liverpool, UK; b Department of Neuroradiology, The Walton Centre NHS Foundation Trust, Liverpool, UK; c Department of Epileptology, University of Bonn, Germany; d Department of NeuroCognition/Imaging, Life&Brain Research Center, Bonn, Germany; e Department of Basic and Clinical Neuroscience, Institute of Psychiatry, Psychology and Neuroscience, King's College London, UK

## Abstract

**Purpose:**

A detailed understanding of white matter tract alterations in patients with temporal lobe epilepsy (TLE) is important as it may provide useful information for likely side of seizure onset, cognitive impairment and postoperative prognosis. However, most diffusion-tensor imaging (DTI) studies have relied on manual reconstruction of tract bundles, despite the recent development of automated techniques. In the present study, we used an automated white matter tractography analysis approach to quantify temporal lobe white matter tract alterations in TLE and determine the relationships between tract alterations, the extent of hippocampal atrophy and the chronicity and severity of the disorder.

**Methods:**

We acquired preoperative T1-weighted and DTI data in 64 patients with well-characterized TLE, with imaging and histopathological evidence of hippocampal sclerosis. Identical acquisitions were collected for 44 age- and sex-matched healthy controls. We employed automatic probabilistic tractography DTI analysis using TRActs Constrained by UnderLying Anatomy (TRACULA) available in context of Freesurfer software for the reconstruction of major temporal lobe tract bundles. We determined the factors influencing probabilistic tract reconstruction and investigated alterations of DTI scalar metrics along white matter tracts with respect to hippocampal volume, which was automatically estimated using Freesurfer's morphometric pipelines. We also explored the relationships between white matter tract alterations and duration of epilepsy, age of onset of epilepsy and seizure burden (defined as a function of seizure frequency and duration of epilepsy).

**Results:**

Whole-tract diffusion characteristics of patients with TLE differed according to side of epilepsy and were significantly different between patients and controls. Waypoint comparisons along each tract revealed that patients had significantly altered tissue characteristics of the ipsilateral inferior-longitudinal, uncinate fasciculus, superior longitudinal fasciculus and cingulum relative to controls. Changes were more widespread (ipsilaterally and contralaterally) in patients with left TLE while patients with right TLE showed changes that remained spatially confined in ipsilateral tract regions. We found no relationship between DTI alterations and volume of the epileptogenic hippocampus. DTI alterations of anterior ipsilateral uncinate and inferior-longitudinal fasciculus correlated with duration of epilepsy (over and above effects of age) and age at onset of epilepsy. Seizure burden correlated with tissue characteristics of the uncinate fasciculus.

**Conclusion:**

This study shows that TRACULA permits the detection of alterations of DTI tract scalar metrics in patients with TLE. It also provides the opportunity to explore relationships with structural volume measurements and clinical variables along white matter tracts. Our data suggests that the anterior temporal lobe portions of the uncinate and inferior-longitudinal fasciculus may be particularly vulnerable to pathological alterations in patients with TLE. These alterations are unrelated to the extent of hippocampal atrophy (and therefore potentially mediated by independent mechanisms) but influenced by chronicity and severity of the disorder.

## Highlights

• We investigated white matter tract alterations in TLE using an automated tool.
• Patients showed bilateral tract alterations but were more prominent ipsilaterally.
• Along-the-tract analyses revealed the exact location of tract diffusion alterations.
• Alterations correlated with chronicity/severity of TLE but not hippocampal atrophy.

## 1 Introduction

Epilepsy is the most common serious neurological disorder with a prevalence estimated to be approximately 1% of the general population (World Health Organization, 2016, Neligan et al., 2012, Ngugi et al., 2010). Temporal lobe epilepsy (TLE) is the most frequent form of medically refractory focal epilepsy (Almeida et al., 2012) and is potentially amenable to surgical resection (Wiebe et al., 2001). Hippocampal atrophy is the most common neuropathological correlate of TLE and the preoperative detection of hippocampal atrophy on magnetic resonance imaging (MRI) is related to improved postoperative prognosis after temporal lobectomy (Spencer et al., 2005). Extrahippocampal abnormalities have been frequently described in TLE, including cortical and subcortical gray matter alterations demonstrated using morphometric techniques (see reviews by Bernhardt et al., 2013, Keller and Roberts, 2008, Richardson, 2012, Bonilha and Keller, 2015) and white matter tract alterations using diffusion tensor imaging (DTI) and tractography (see reviews by Rodríguez-Cruces and Concha, 2015, Gross, 2011). Tractography approaches have proved to be particularly interesting as they can provide quantitative information regarding neuroanatomical structure and pathology that exist beyond conventional visual analysis of MRI scans, and may yet provide important information on cognitive deficits and treatment prognosis in people with epilepsy. Furthermore, the recent revision of seizure disorder definitions to acknowledge the importance of networks for the onset of focal seizures (Berg and Scheffer, 2011) has encouraged a new direction of imaging research to model neuroimaging data in context of structural and functional networks and connectivity (Richardson, 2012). Reconstruction of white matter tracts from DTI data represent the most frequently applied technique of generating structural connectivity in the human brain (Jellison et al., 2004, Mori et al., 2009).

There are a variety of ways to reconstruct white matter tracts from DTI data. The most frequently applied have been manual tractography techniques that require a trained researcher to segment individual tract bundles based on known anatomical features. Using these approaches, previous studies have reported significant alterations in DTI scalar metrics, such as fractional anisotropy (FA) and mean diffusivity (MD) of the parahippocampal fibers (Ahmadi et al., 2009, Yogarajah et al., 2008, Concha et al., 2007), inferior (Ahmadi et al., 2009, Imamura et al., 2015, Concha et al., 2012) and superior (Ahmadi et al., 2009, Concha et al., 2012, Lin et al., 2008) longitudinal fasciculus, uncinate fasciculus (Ahmadi et al., 2009, Rodrigo et al., 2007, Diehl et al., 2008, Lin et al., 2008, Concha et al., 2012) and fornix (Concha et al., 2005, Concha et al., 2007, Concha et al., 2010). However, a significant practical limitation of manual tractography methods is the availability of trained personnel and the time consuming nature of making measurements. Similar to advantages associated with the evolution of automated morphometric techniques from manual volumetric approaches based on T1-weighted (T1-w) MRI data (Bonilha and Keller, 2015), a technique that automatically reconstructs probabilistic white matter tract bundles could circumnavigate some of the shortcomings of manual tractography approaches.

Freesurfer software (Fischl, 2012) has continued to develop and provide freely available tools along with community support for morphometric and tractographic analyses. In context of this software, TRActs Constrained by UnderLying Anatomy (TRACULA, Yendiki et al., 2011) offers the opportunity to automatically reconstruct major white matter bundles. A significant advantage of this approach is that the automated reconstruction of tracts is performed in each subject's native space without warping to standard space and tissue characteristics of white matter tracts can be investigated in relation to morphometric analyses of subcortical and cortical structures (Storsve et al., 2016, Sølsnes et al., 2016). This is particularly interesting in TLE with associated hippocampal sclerosis as relationships between extent of damage to the epileptogenic hippocampus and tract tissue characteristics can be investigated. Hippocampal sclerosis may occur in response to an initial precipitating injury prior to a period of epileptogenesis that later gives rise to the onset of recurrent spontaneous seizures (Pitkänen and Lukasiuk, 2011, Blume, 2006, Goldberg and Coulter, 2013). However, the etiology of extrahippocampal brain alterations in TLE remains unknown. Some morphometric MRI studies have suggested that the extent of hippocampal atrophy is correlated with the degree of extrahippocampal temporal lobe atrophy (Moran et al., 2001, Bonilha et al., 2010, Mueller et al., 2009, McMillan et al., 2004), suggesting that a common process of gross atrophy may occur. However, less is known about the relationship between hippocampal atrophy and white matter tract alterations (Rodríguez-Cruces and Concha, 2015). One study revealed a correlation between the extent of hippocampal atrophy and white matter tract alterations (Scanlon et al., 2013) but another study reported no relationship (Concha et al., 2012). In the present study, we investigated the relationships between extent of hippocampal atrophy, as quantified using Freesurfer morphometric tools, temporal lobe white matter tract alterations, and various clinical aspects of TLE.

There is only limited anatomical specificity provided in conventional tractography studies in TLE, as techniques are typically restricted to the analysis of whole-tract diffusion alterations. Information obtained from whole-tract analyses are limited because there may be significant variations in diffusion characteristics along the length of white matter tracts (Johnson et al., 2013), and it is likely that some pathological tract alterations occur in circumscribed regions within tracts and not along entire tracts in patients with TLE. Therefore it is important to develop methods that permit analysis of within-tract tissue characteristics in patients with TLE (Concha et al., 2012, Glenn et al., 2016). In the present study, we sought to apply TRACULA methods to investigate within-tract alterations in TLE, and to determine whether these regional alterations are influenced by the extent of hippocampal atrophy and clinical variables. Despite the potential advantages of utilizing automated tractography approaches, TRACULA has only recently been used in an increased number of clinical studies, including schizophrenia (Yendiki et al., 2011), bipolar disorder (Sprooten et al., 2016), myotonic dystrophy (Wozniak et al., 2014) and Alzheimer's disease (Lee et al., 2015). Despite the increasing application of tract analysis techniques in neurological and neuropsychiatric disorders, there have been no applications of TRACULA to date in epilepsy, which is characterized by regional gray and white matter pathology.

## 2 Methods

### 2.1 Participants

We studied 64 patients with well-characterized TLE and associated ipsilateral hippocampal sclerosis (41 patients with left TLE, 23 patients with right TLE) and 44 age- and sex-matched controls. Age and sex did not differ significantly between patients and controls and we found no significant difference in any of the clinical variables (*p* > 0.05) between patients with left and right TLE (Table 1). Radiological evidence of hippocampal sclerosis was assessed by an experienced neuroradiologist using standard criteria, including hippocampal volume loss and internal structural disruption on T1-weighted (T1-w) MRI and/or hyperintensities on T2-weighted/FLAIR images (Keller et al., 2015a, Keller et al., 2015b). There was no evidence of bilateral hippocampal sclerosis in any patient or of a secondary extrahippocampal lesion that may have contributed to seizures (Keller et al., 2015a). All patients underwent comprehensive pre-surgical evaluation, and all had a confident diagnosis of mesial TLE based on semiological, electrophysiological and imaging investigations (Kral et al., 2002). All patients underwent amygdalohippocampectomy and hippocampal sclerosis was confirmed histologically using standard criteria (Blumcke et al., 2013). All patients and controls provided written informed consent and the local ethics committee approved this study.

**Table 1:** Demographic and clinical variables according to seizure laterality.

	Group			Stats	
	Left TLE	Right TLE	Controls	Statistic	*p*-Value
N	41 (38%)	23 (21%)	44 (41%)	–	–
Sex (female/male)	26/15	10/13	29/15	*χ*^2^(2) = 3.5	0.2
Febrile seizures (no/yes)	20/8	10/5	–	*χ*^2^(1) = 0.1	0.8
SGTCS (no/yes)	15/13	9/6	–	*χ*^2^(1) = 0.2	0.7
Variables with mean (standard deviation)					
Age (years)	43.8 (13)	41.3 (14.9)	43.2 (14.2)	*F*(2,107) = 0.24	0.8
Age at onset in years	18 (12.3)	14.3 (11.5)	–	*T*(62) = 1.2	0.3
Duration in years	22.7 (13.1)	22 (17.5)	–	*T*(62) = 0.17	0.9
Seizure frequency in one month	5.4 (3.9)	9.4 (22.4)	–	*T*(41) = − 0.9	0.4
Seizure burden	1.9 (0.45)	1.8 (0.44)	–	*T* (41) = 0.9	0.4

*Note*. Abbreviation: TLE = temporal lobe epilepsy. The conducted statistical tests were Chi-squared tests of independence for the first three variables; one-way ANOVA for age and unpaired two-tailed *t*-tests for all remaining clinical variables.

### 2.2 MR acquisition

Each participant underwent MRI at the Life & Brain Center in Bonn on a 3 Tesla scanner (Magnetom Trio, Siemens, Erlangen, Germany). 3D T1-w (MPRAGE, 160 slices, repetition time (TR) = 1300 ms, inversion time (TI) = 650 milliseconds, echo time (TE) = 3.97 ms, resolution = 1.0 × 1.0 × 1.0 mm, flip angle = 10) and DTI (diffusion-weighted single shot spin-echo planar imaging sequence, TR = 12 s, TE = 100 ms, 72 axial slices, resolution = 1.726 × 1.726 × 1.7 mm, no cardiac gating, GRAPPA acceleration factor = 2.0, 60 images with diffusion weighting, b = 1000 s/mm^2^, seven images without diffusion-weighting, b = 0 s/mm^2^, subsequently referred to as b0 images) scans were acquired for all participants.

### 2.3 Data pre-processing

For each participant we performed automated segmentation and cortical parcellation of T1-w data using Freesurfer version 5.3.0. The standard Freesurfer “recon-all” processing stream was used, which provides surfaces and morphometry data for each subject (Keller et al., 2012) in addition to gray and white matter segmentations. This information was also subsequently used to restrict tractography analysis to white matter. However, before tensor fitting and tractography were performed within Freesurfer (TRACULA version 1.56), DTI data was processed using the ENIGMA DTI-preprocessing steps (http://enigma.ini.usc.edu/protocols/dti-protocols/). In particular, we used the first b0 image as a reference for co-registration of subsequent b0 images (FSL FLIRT, Smith et al., 2004). The resulting co-registered b0 images were averaged and served as a reference image during motion correction on the diffusion-weighted images. The gradient table information was adjusted accordingly (Leemans and Jones, 2009). Subsequently the data was processed in order to account for geometric distortions, this was performed on the mean b0 image via the T1-w scan. In order to achieve distortion correction, the T1-w scan was rigidly aligned with the mean b0 image (Smith et al., 2004) and the mean b0 image was nonlinearly registered to this T1-w scan in diffusion space using Advanced Normalization Tools (http://stnava.github.io/ANTs/). The resulting nonlinear registration information was used to unwarp subsequent diffusion-weighted images in native diffusion space. TRACULA's default tensor fitting and tract reconstruction pipelines using the ball-and-stick model were applied to the pre-processed data.

### 2.4 Quality assessment after pre-processing

The DTI data, T1-w Freesurfer segmentations and anatomical alignment of the co-registered b0/T1 images were assessed visually. We visually appraised TRACULA's performance in terms of tract reconstruction for the data that had been processed without using the T1-w image for EPI-distortion correction and recorded any failed reconstructions. We used the documented reinitialization step in order to recover the tracts.

As DTI-derived scalar metrics have been shown to vary with Signal-to-Noise-Ratio (SNR) (Farrell et al., 2007), we calculated the SNR for every diffusion-weighted image through TRACULA using the mean of the signal intensity of the whole brain white matter divided by the standard deviation of the same area. We averaged all SNR values for each participant for further statistical analysis. As all SNR values are derived from whole-brain images acquired on the same scanner, variability is expected to be low and normally distributed. We tested the data for normality and for differences in mean SNR of all three groups using a one-way ANOVA (data normally distributed, Lilliefors *p* > 0.05).

Diffusion metrics have been shown to be vulnerable to differences in motion across patient-control cohorts. We therefore assessed any differences in the total motion index (TMI) between patients with left and right TLE and controls. The TMI has been previously defined as a summary value which takes average translation/rotation, slice dropout and dropout severity into account and may reduce the number of false positives when used as a nuisance regressor during analysis of data collected on study participants who have moved differently (Yendiki et al., 2013). We tested the data for normality and for differences in mean TMI of all three groups using a Kruskal-Wallis ANOVA (data non-normally distributed, Lilliefors < 0.05). Multiple comparison correction was performed using the Dunn-Sidak approach (Dunn, 1964).

### 2.5 Extraction of volumes and tracts

**Fig. 1:**
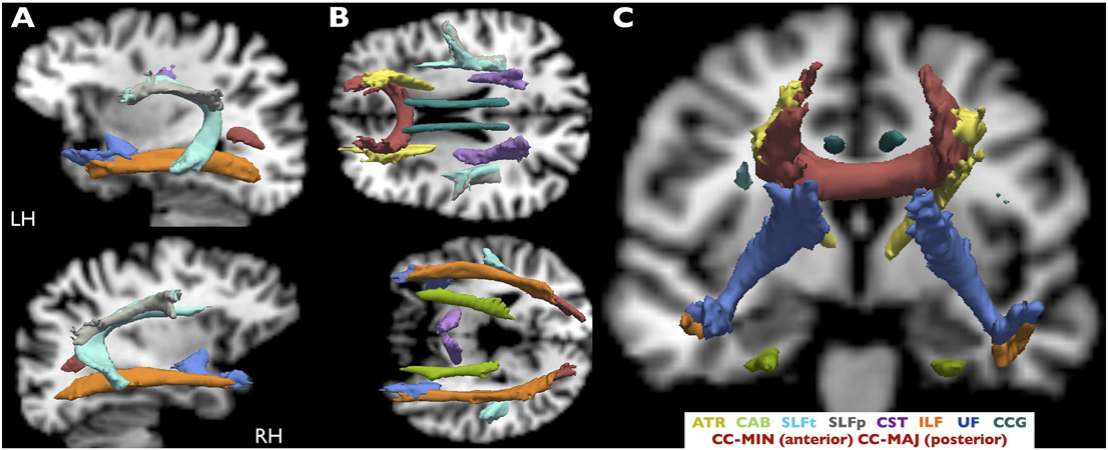
All reconstructed TRACULA tracts. Estimated probability tracts from TRACULA are overlaid on a healthy subject's T1-w scan (native space) and shown in sagittal (A), axial (B) and coronal (C) views. TRACULA tracts included in further analysis were CAB, SLFt, ILF and UF. All images are presented in radiological convention. RH = Right Hemisphere; LH = Left Hemisphere; CC-MIN: corpus callosum – forceps minor; CC-MAJ: corpus callosum – forceps major; ATR: anterior thalamic radiations; UF: uncinate fasciculus; ILF: inferior longitudinal fasciculus; CAB: cingulum–angular bundle; SLFt: superior longitudinal fasciculus - temporal segment; SLFp: superior longitudinal fasciculus - parietal segment; CST: corticospinal tract; CCG: cingulum–cingulate gyrus bundle. All tracts are shown in isosurface at 20% of maximum probability.

For patients and controls we extracted intracranial volume (ICV), left and right hippocampal volumes from Freesurfer's recon-all segmentation step (Table 2). Subsequently we investigated tract DTI-derived scalar metrics (fractional anisotropy (FA) and mean diffusivity (MD)) for tracts previously reported to be affected in patients with TLE using manual tractography techniques. The following temporal lobe tracts were analyzed: the temporal segment of the superior longitudinal fasciculus (SLFt), inferior longitudinal fasciculus (ILF), uncinate fasciculus (UF) and cingulum angular bundle (CAB; otherwise referred to as the parahippocampal white matter bundle) (Fig. 1). Unfortunately TRACULA does not provide tract reconstruction of the fornix, we were therefore unable to investigate this structure in the present study. TRACULA directly provides tract mean DTI-derived metric values. Value extraction was confined to the center of the tract for all tracts investigated (via the most probable single fiber pathway).

**Table 2:** Kruskal-Wallis ANOVA comparing volumes across all groups.

	Side	M (SD) in mm^3^			Comparisons (corrected *p*-values)			Stats	
		lTLE	C	rTLE	lTLE vs C	lTLE vs rTLE	C vs rTLE	Chi^2^-statistic	*p*-Value
Intracranial volume (ICV)	Whole brain	1,504,800 (261470)	1,645,200 (122970)	1,568,100 (23783)	< 0.05	0.79	0.47	6.6	< 0.05
Hippocampal volume corrected for ICV (%)	Left	0.2 (0.06)	0.27 (0.02)	0.27 (0.03)	< 0.001	< 0.001	0.9	35.2	< 0.001
	Right	0.3 (0.05)	0.27 (0.02)	0.2 (0.04)	0.9	< 0.001	< 0.001	40.5	< 0.001

*Note*. Abbreviations: M = mean; SD = standard deviation; lTLE = left temporal lobe epilepsy; C = control; rTLE = right temporal lobe epilepsy.

### 2.6 Statistical analysis

For all tests, results were considered significant at *p* < 0.05. Statistical testing was performed using a Macintosh Laptop OSX 10.9.2 running MATLAB R2015b. As native space volumes and tracts cannot be assumed to be normally distributed due to inter-subject variability we tested the normality of the data. Given that our data pertaining to hippocampal/intracranial volumes and DTI-derived metric values were non-normally distributed (Lilliefors *p* < 0.05), we used non-parametric tests for analysis. Patient-control group comparisons on hippocampal volumes/ICV were performed using a Kruskal-Wallis ANOVA, with a Dunn-Sidak correction for multiple comparisons (Dunn, 1964). Patient-control group comparisons on mean tract diffusion metrics were performed using a non-parametric ANCOVA with a single regression model which accounted for both multiple comparisons and TMI (Conover and Iman, 1982). Additionally we grouped the patients dichotomously according to presence of childhood febrile convulsions and secondary-generalized tonic-clonic seizures (SGTCS) and investigated differences in DTI-metrics using the unpaired Wilcoxon-rank-sum test.

TRACULA offers a DTI-metric value along the trajectory of a pathway through generating a weighted average of the respective values over all sampled paths for a particular tract. As native space tracts had a slightly different length for each data set and the endings did not always correspond, shorter tracts contained some NaN (‘not a number’) waypoints, which were ignored in statistical testing. This comparison was based solely on native space tract values and did not represent a voxel-based approach, where each voxel represents the same type of region of tissue after normalization to standard space. However, in order to visualize differences between patients and controls along the different tracts, mean tract paths in MNI space were generated. Waypoint average FA/MD values along the native space tracts were separately compared between patients with left/right TLE and controls using a non-parametric ANCOVA and a single regression model which accounted for both multiple comparisons and TMI. The unpaired Wilcoxon-Rank-Sum test was performed on patients with and without history of childhood febrile convulsions/SGTCS. As multiple locations along a tract were analyzed, significance levels were corrected with the False-Discovery-Rate (FDR) procedure by Benjamini and Hochberg, 1995 and considered significant at *p* < 0.05.

Correlations between whole-tract and waypoint DTI-metrics with extent of hippocampal atrophy and clinical information were investigated using Pearson product-moment linear correlation coefficients. Clinical information included age, age at onset of epilepsy, duration of epilepsy and seizure burden. Hippocampal volume was corrected for ICV and duration was corrected for patient age to control for the effects of normal brain maturation. Seizure burden was defined as log10(*frequency* × *duration*). The logarithm was applied in order to accommodate patients with very high seizure frequency. Significance levels were corrected with the FDR procedure and considered significant at *p* < 0.05.

## 3 Results

### 3.1 Data quality assessment

Visual assessment revealed satisfactory T1-w Freesurfer segmentations and excellent anatomical agreement between b0 and T1-w images after co-registration. Using the conventional image processing stream for TRACULA at least one tract was only partially reconstructed in 50 participants (46%). However, all tracts were successfully reconstructed after reinitialization.

No significant difference between groups in terms of SNR were found (left TLE: mean = 4.02, std = 0.22; right TLE: mean = 4.03, std = 0.21; controls: mean = 3.94, std = 0.19); *F*(2,107) = 2.15 *p* = 0.12. However, we did find that the TMI was significantly different between patients with left TLE (mean TMI = − 0.6, SD = 0.95), right TLE (mean TMI = − 0.7, SD = 0.99) and controls (mean TMI = 1.2, SD = 0.99) (*χ*^2^(2,107) = 52.1 *p* = 4.9 ∗ 10^− 12^). Post hoc testing revealed that controls had a higher TMI than either patient group (*p* < 0.001), whereas the two patient groups did not differ significantly among each other (*p* = 0.9). The TMI value was entered as a nuisance regressor into the non-parametric ANCOVA in order to mitigate confounding effects on patient-control comparisons (Yendiki et al., 2013).

### 3.2 Patients vs controls

#### 3.2.1 Volumes

There were no differences in ICV between patient groups. However, ICV was significantly smaller for patients with left TLE relative to controls (Table 2). Patients with left TLE and right TLE had significantly smaller ipsilateral hippocampal volumes (corrected for ICV) than controls. There was no evidence of contralateral hippocampal atrophy in patients relative to controls. None of the demographic (age) or clinical variables (age at onset, duration corrected for age and seizure burden) correlated with ipsilateral hippocampal volumes (corrected for ICV).

#### 3.2.2 Whole-tract diffusion metric analysis

**Fig. 2:**
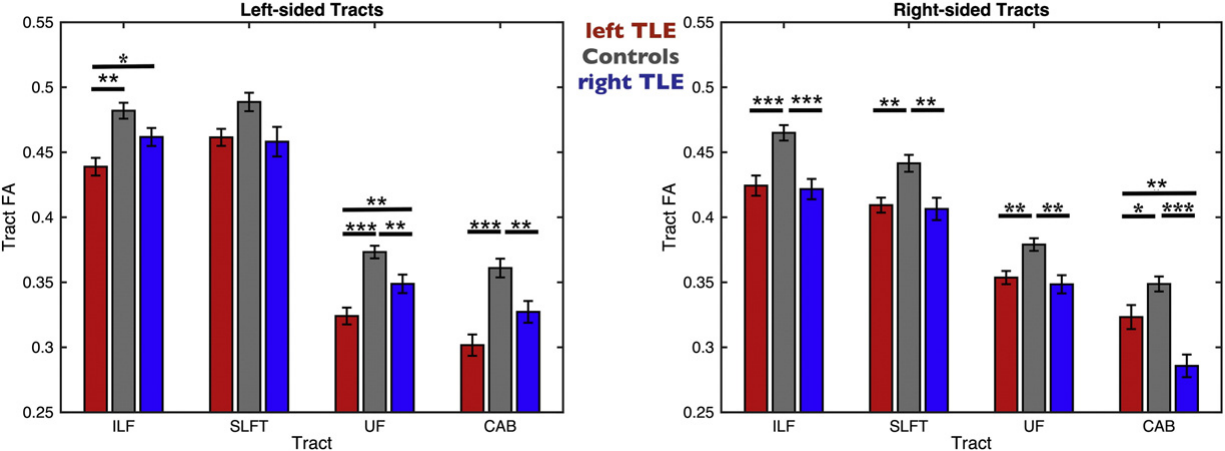
FA Values from TRACULA Tracts ILF, SLFt, UF and CAB. The plot shows mean center tract FA distributions along with error bars for left and right tracts of patients with left TLE (red bars), controls (gray bars) and patients with right TLE (blue bars). Asterisks and bars show significantly reduced FA values for patients when comparing to controls and between the two patient groups. *p < 0.05; **p < 0.01; ***p < 0.001.

Whole-tract FA values are presented in Fig. 2 and ﻿Table 3.﻿ Patients with left TLE had a significant decrease in FA of all tracts (except ipsilateral SLFT) in both hemispheres compared to controls. Relative to controls, patients with right TLE had a significant decrease in FA in all ipsilateral tracts, and a decrease in FA in contralateral UF and CAB. Additionally we found a significant decrease in FA in the left UF and ILF in patients with left TLE relative to patients with right TLE, and a significant decrease in FA in patients with right TLE in the right CAB relative to patients with left TLE. Across all patients, whole-tract FA values did not correlate with ipsilateral hippocampal volumes. There were significant correlations between patient age and FA in the ipsilateral (rho = − 0.4, *p* < 0.01) and contralateral (rho = − 0.26, *p* < 0.05) UF and the contralateral SLFt (rho = − 0.36, *p* < 0.01). Duration of epilepsy (corrected for age) was significantly correlated with FA in the contralateral UF (rho = − 0.27, *p* < 0.05). Seizure burden was significantly correlated with FA values in the ipsilateral (rho = − 0.33, *p* < 0.05) and contralateral (rho = − 0.32, *p* < 0.05) UF. Age at onset of epilepsy and seizure frequency did not correlate with FA in any tract.

**Table 3:** Non-parametric ANCOVA comparing FA across all groups.

Tract	Side	M (SD)			*p*-Values from regression model			Stats	
		lTLE	C	rTLE	lTLE vs C	lTLE vs rTLE	C vs rTLE	F-statistic	*p*-Value
SLFt	Left	0.46 (0.04)	0.49 (0.05)	0.46 (0.05)	0.2	0.9	0.33	4	< 0.05
	Right	0.41 (0.04)	0.44 (0.04)	0.41 (0.04)	< 0.01	0.69	< 0.01	7.05	< 0.01
ILF	Left	0.44 (0.04)	0.48 (0.04)	0.46 (0.03)	< 0.01	< 0.05	0.29	4.06	< 0.05
	Right	0.42 (0.05)	0.46 (0.04)	0.42 (0.04)	< 0.001	0.59	< 0.001	11.78	< 0.001
UF	Left	0.32 (0.04)	0.37 (0.03)	0.35 (0.03)	< 0.001	< 0.01	< 0.01	16.5	< 0.001
	Right	0.35 (0.03)	0.38 (0.03)	0.35 (0.03)	< 0.01	0.68	< 0.01	7.01	< 0.01
CAB	Left	0.3 (0.05)	0.36 (0.05)	0.33 (0.04)	< 0.001	0.09	< 0.01	6.2	< 0.01
	Right	0.32 (0.06)	0.35 (0.04)	0.29 (0.04)	< 0.05	< 0.01	< 0.001	15.2	< 0.001

*Note*. Abbreviations: M = mean; SD = standard deviation; lTLE = left temporal lobe epilepsy; C = control; rTLE = right temporal lobe epilepsy; FA = fractional anisotropy; SLFt = superior longitudinal fasciculus (temporal segment); ILF = inferior longitudinal fasciculus; UF = uncinate fasciculus; CAB = cingulum angular bundle.

**Fig. 3:**
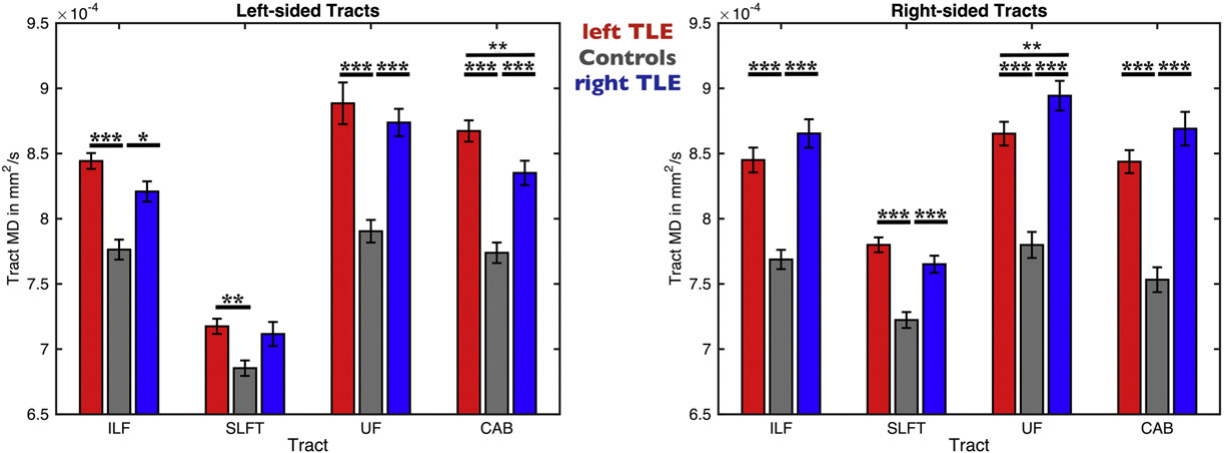
MD Values from TRACULA Tracts ILF, SLFt, UF and CAB. The plot shows mean center tract MD distributions along with error bars for left and right tracts of patients with left TLE (red bars), controls (gray bars) and patients with right TLE (blue bars). Asterisks and bars show significantly reduced MD values for patients when comparing to controls. *p < 0.05; **p < 0.01; ***p < 0.001.

Whole-tract MD values are presented in Fig. 3 and Table 4. Larger patient-control differences were observed in MD relative to FA. Patients with left and right TLE had bilateral increase in MD of all tracts (except for left SLFT in patients with right TLE) relative to controls. Additionally we found increased MD in the left CAB in patients with left TLE relative to patients with right TLE and increased MD in the right UF in patients with right TLE relative to patients with left TLE. Whole-tract MD values did not correlate with ipsilateral hippocampal volume. Age correlated with the MD of the ipsilateral SLFt (rho = 0.35, *p* < 0.01). Duration of epilepsy (corrected for age) was significantly correlated with MD in the ipsilateral (rho = 0.3, *p* < 0.05) and contralateral (rho = 0.46, *p* < 0.001) UF and with the contralateral ILF (rho = 0.3, *p* < 0.05). Seizure burden was significantly correlated with MD in the contralateral UF (rho = 0.31, *p* < 0.05). Age at onset of epilepsy was significantly correlated with MD of the contralateral UF (rho = − 0.35, *p* < 0.01). Seizure frequency did not correlate with any diffusion measure. There were no significant differences in FA or MD of any tract between patients with/without a history of childhood febrile convulsions, or between those with/without secondary seizure generalization (*p* > 0.05).

**Table 4:** Non-parametric ANCOVA comparing MD across all groups.

Tract	Side	M(SD) (in 10^–4^ mm^2^/s)			*p*-Values from regression model			Stats	
		lTLE	C	rTLE	lTLE vs C	lTLE vs rTLE	C vs rTLE	*F*-statistic	*p*-Value
SLFt	Left	7.2 (0.4)	6.9 (0.4)	7.1 (0.4)	< 0.01	0.5	0.07	6.5	< 0.01
	Right	7.8 (0.4)	7.2 (0.4)	7.7 (0.3)	< 0.001	0.17	< 0.001	20.5	< 0.001
ILF	Left	8.4 (0.4)	7.8 (0.5)	8.2 (0.4)	< 0.001	0.28	< 0.05	17.24	< 0.001
	Right	8.4 (0.6)	7.7 (0.5)	8.7 (0.5)	< 0.001	0.06	< 0.001	41.8	< 0.001
UF	Left	8.9 (1)	7.9 (0.6)	8.7 (0.5)	< 0.001	0.74	< 0.001	18.9	< 0.001
	Right	8.7 (0.6)	7.8 (0.7)	8.9 (0.5)	< 0.001	< 0.05	< 0.001	33.02	< 0.001
CAB	Left	8.7 (0.5)	7.7 (0.5)	8.4 (0.4)	< 0.001	< 0.05	< 0.001	26.8	< 0.001
	Right	8.4 (0.6)	7.5 (0.6)	8.7 (0.6)	< 0.001	0.14	< 0.001	38.2	< 0.001

*Note*. Abbreviations: M = mean; SD = standard deviation; lTLE = left temporal lobe epilepsy; C = control; rTLE = right temporal lobe epilepsy; FA = fractional anisotropy; SLFt = superior longitudinal fasciculus (temporal segment); ILF = inferior longitudinal fasciculus; UF = uncinate fasciculus; CAB = cingulum angular bundle.

#### 3.2.3 Waypoint diffusion metric analysis

**Fig. 4:**
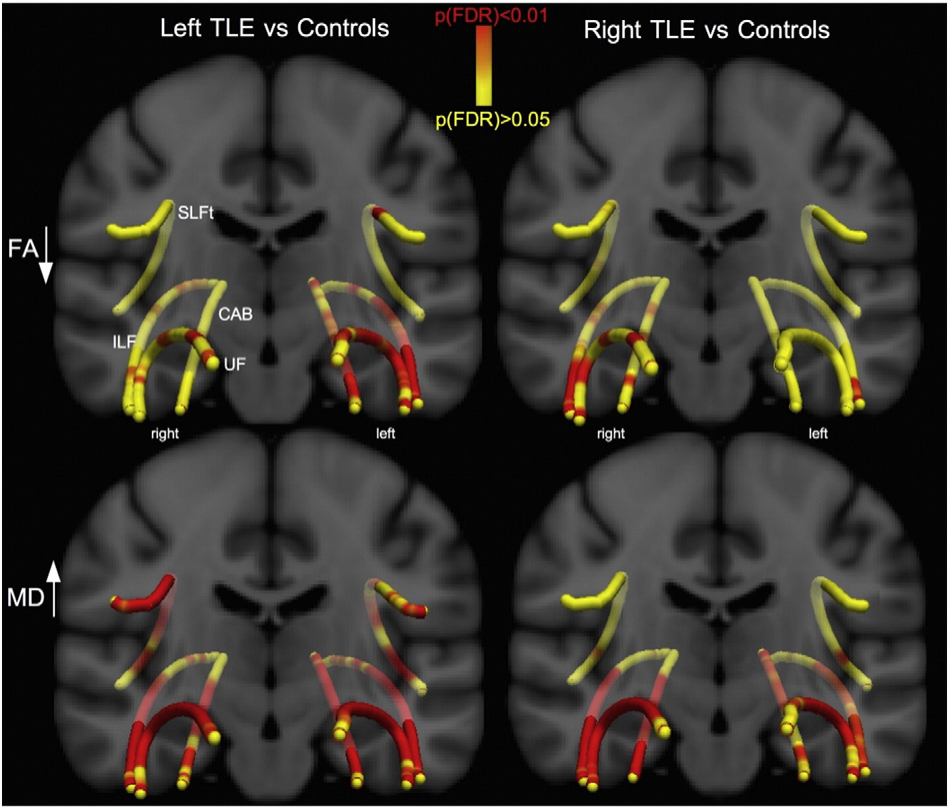
Waypoint comparison *p*-values along the tracts. Differences between the patient groups and controls are shown projected onto a T1-w template. Red regions show significantly reduced FA (first row) and increased MD (second row) relative to controls. Changes are more pronounced in MD than in FA and patients with left TLE are more bilaterally affected than patients with right TLE. TLE = temporal lobe epilepsy; FA = fractional anisotropy; MD = mean diffusivity; SLFt = superior longitudinal fasciculus - temporal segment; CAB = cingulum–angular bundle; ILF = inferior longitudinal fasciculus; UF = uncinate fasciculus.

**Fig. 5:**
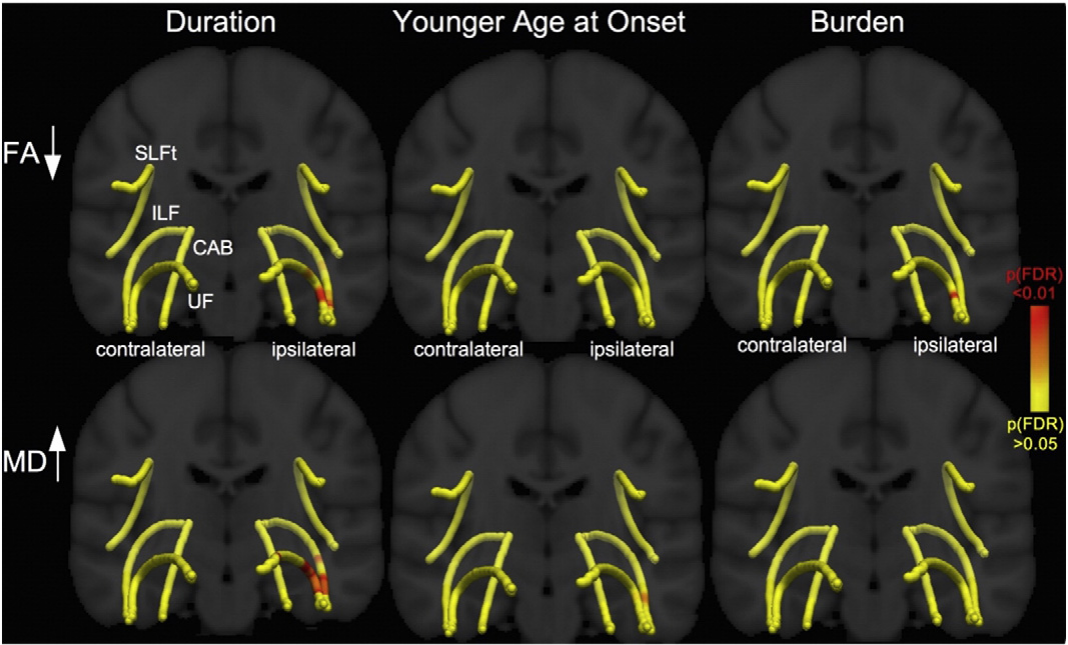
Waypoint correlation *p*-values along the tracts. Relationships between the DTI-metrics and clinical variables are shown projected onto a T1-w template and mean tract pathways. Red regions show significant correlations with reduced FA (first row) and increased MD (second row). Relationships between duration (corrected for age) and FA/MD of ipsilateral anterior UF and ILF regions and correlations between age at onset (MD of UF/ILF) and seizure burden (FA of UF) were found. FA = fractional anisotropy; MD = mean diffusivity; SLFt = superior longitudinal fasciculus - temporal segment; CAB = cingulum–angular bundle; ILF = inferior longitudinal fasciculus; UF = uncinate fasciculus.

Waypoint comparisons along the native tracts are shown in Fig. 4 and revealed regionally reduced FA within the UF, ILF and CAB, predominantly ipsilateral to seizure onset, with fewer widespread changes in contralateral tracts. For both patient groups tract alterations were more widespread in analysis of MD and affected ipsi- and contralateral tracts. For both FA and MD, patients with left TLE showed more extensive bilateral changes (FA of contralateral ILF/UF, MD of SLFt) than patients with right TLE (only a small region affected in FA of contralateral ILF, MD abnormalities were not observed in at least a third of the length of contralateral tracts). Along-the-tract DTI-metrics and extent of ipsilateral hippocampal atrophy were not correlated. There were significant correlations between patient age and FA of the bilateral UF/SLFt and MD contralaterally. Correlations between FA/MD and clinical variables are shown in Fig. 5. Duration of epilepsy was significantly correlated with FA and MD in anterior temporal sections of UF and ILF. Younger age at onset of TLE did not correlate with FA, but there was a significant correlation with MD in the anterior temporal lobe portions of the ipsilateral UF and ILF. Seizure burden was significantly correlated with FA in anterior regions of the ipsilateral UF. Seizure frequency did not correlate with any diffusion measure. There were no significant differences in FA or MD along any tract between patients with/without a history of childhood febrile convulsions, or between those with/without secondary seizure generalization (*p* > 0.05).

## 4 Discussion

There were three primary objectives of the present study. Firstly, we sought to investigate diffusion alterations of whole temporal lobe tracts in patients with TLE relative to healthy controls using an automated tractography approach. We found that whole-tract FA/MD abnormalities were observed in nearly all temporal lobe tracts investigated, the effects being observed bilaterally, but most strongly ipsilaterally. Tract diffusion alterations were more strongly bilaterally distributed in patients with left TLE. Secondly, we investigated within-tract alterations using waypoint comparisons, which confirmed that ipsilateral tracts were more extensively affected than contralateral tracts and additionally identified specific regions within tracts that demonstrate alterations in diffusion characteristics. Thirdly, we sought to investigate the relationship between regional tract alterations, the degree of the hippocampal atrophy, and the chronicity and severity of TLE. We found that extent of hippocampal atrophy was not related to (i) the degree of FA and MD alterations of temporal lobe tracts or (ii) the clinical characteristics of patients, whereas diffusion alterations of ipsilateral temporal lobe tracts were significantly related to age at onset of epilepsy, duration of epilepsy and epilepsy burden. We discuss the biological and clinical implications of this work before highlighting pertinent methodological issues.

### 4.1 Biological and clinical implications

Consistent with previous studies investigating diffusion characteristics of the ILF, SLF, cingulum and UF using manual DTI tractography approaches (Ahmadi et al., 2009, Yogarajah et al., 2008, Rodrigo et al., 2007, Diehl et al., 2008, Lin et al., 2008), we demonstrate significant alterations in bihemispheric temporal lobe tract diffusion characteristics in patients with unilateral TLE using the fully automated TRACULA tractography pipelines. Diffusion measurement analysis revealed that patients with left TLE are more bilaterally affected than patients with right TLE (Ahmadi et al., 2009, Keller et al., 2012, Kemmotsu et al., 2011) and waypoint comparisons localized these changes more precisely. However, it should be noted that other studies have reported increasingly bilateral brain alterations in patients with right TLE (Garcıa-Fiñana et al., 2006, Pail et al., 2010).

FA and MD have proved to be useful in characterizing microstructural brain damage in patients with TLE. However, FA is a measure of microstructural integrity computed through a normalized ratio of radial/axial diffusivities (Alexander et al., 2007, Soares et al., 2013) and could therefore imply multiple microstructural changes (e.g. demyelination, loss of axons, increased inter-axonal space). MD, as a directionally averaged value that takes all diffusivity measures into equal account, may demonstrate the magnitude of fluid viscosity and is sensitive to cellularity, edema and necrosis (Alexander et al., 2011). Therefore, MD may potentially permit inferences about microstructure in terms of loss of axon membrane and thus provide in-vivo measurements that strongly relate to histopathology. In fact, a recent study on correlations between diffusion alterations and histology found that preoperative diffusion alterations were related to an increase in extra-axonal fraction, reduced cumulative axonal membrane circumference and myelin of the resected tissue (Concha et al., 2010). Interestingly, we found that MD is more sensitive than FA in determining differences between patients versus controls and is able to detect extensive bilateral effects. These findings are consistent with those reported in other studies that have employed within-tract analyses to demonstrate increased alterations in MD in patients with mesial TLE (Concha et al., 2012, Glenn et al., 2016), although similar findings have not been reported in patients with cryptogenic TLE (Keller et al., 2013). The inconsistencies may in part be explained by different types of analysis approaches, as, for example, a tract-based approach has been shown to be more sensitive than voxel-based ones (Focke et al., 2008). Furthermore, some studies have found that diffusivity measures have a higher spatial correspondence with discharges from stereo-electroencephalography than anisotropy measures (Thivard et al., 2006, Guye et al., 2007), may be superior in detecting occult damage (Rugg-Gunn et al., 2001) and have been linked to dynamics of seizure activity (Yu and Tan, 2008).

In contrast to recent publications using automated whole-tract tractography in standard space (Hagler et al., 2009), and in accordance with other deterministic automated and manual along-the-tract approaches (Glenn et al., 2016, Concha et al., 2012 respectively), we demonstrate that automated probabilistic tractography can detect alterations along temporal lobe tracts in native space. The present study revealed that TRACULA's waypoint analysis is sensitive to within-tract differences, which may have useful diagnostic (e.g. identification of occult damage) and prognostic (e.g. development of imaging biomarkers) applications. Along-the-tract analysis revealed no correlations with ipsilateral hippocampal volume, which is consistent with previously conducted manual deterministic tractography (Concha et al., 2012) and region-of-interest analysis (Keller et al., 2012, Bonilha et al., 2010). However, another study reported that extrahippocampal FA, approximately located in the ipsilateral superior segment of the cingulum and other extratemporal tracts not assessed in the present study, was correlated with hippocampal volume (Scanlon et al., 2013). These latter findings were also reported in the cohort of healthy controls (Scanlon et al., 2013).

An earlier age at onset of epilepsy, longer duration of epilepsy and increased epilepsy burden were related to greater diffusion alterations of temporal lobe white matter tracts, but not the extent of hippocampal atrophy. A correlation between duration of epilepsy and extrahippocampal alterations has been reported in studies (Chiang et al., 2016, Govindan et al., 2008, Lin et al., 2008, Keller et al., 2012). Given the relationships between clinical variables and tissue characteristics along white matter tracts, and the absence of this relationship with hippocampal volume, these findings may suggest that the development of hippocampal and white matter abnormalities are governed by independent developmental mechanisms. The development of mesial TLE is thought to begin with an initial precipitating injury or condition, such as febrile seizures, infection, genetic defects, trauma, stroke or hypoxic damage, which sets in process a period of epileptogenesis. This is a latent period of aberrant neuroplasticity that later supports the onset of spontaneous seizures (Mathern et al., 1996, Pitkänen and Lukasiuk, 2011). It has been suggested that the initial precipitating event, and potentially the years of aberrant hippocampal plasticity, are the primary factors in the development of hippocampal sclerosis, which can be present prior to the onset of habitual seizures (Mathern et al., 1996). This may be the reason why the present study, like several other studies (Cendes et al., 1993, Davies et al., 1996, Keller et al., 2012, Keller et al., 2002) report no relationships between the extent of hippocampal atrophy and age at onset and duration of epilepsy. However, these clinical variables are more significant in the development of extrahippocampal white matter tract abnormalities, which appear to be influenced by the chronicity of TLE and therefore are likely to be secondary in nature. Other studies have reported relationships between diffusion characteristics of white matter and the chronicity of TLE (Glenn et al., 2016, Keller et al., 2012). However, other studies have failed to show a relationship between diffusivity measures and duration of epilepsy (Thivard et al., 2005) or age at onset of epilepsy (Thivard et al., 2005, Keller et al., 2012). The only other study to date investigating correlations between along-the-tract diffusion characteristics and seizure burden reported correlations only with diffusional kurtosis measures of microstructure, but not diffusion tensor measures (Glenn et al., 2016). However, this previous study was performed in standard space, while TRACULA outputs longer tracts in native space, which may account for the differences seen with respect to correlations with seizure burden.

Tractography is already acknowledged by many neurologists and neurosurgeons and increasingly used to perform pre-surgical investigations regarding tract anatomy so that surgical damage to essential brain pathways can be avoided. Furthermore, tractographic analysis may contribute to the understanding of the underlying pathological mechanisms of epilepsy as a network disorder since it allows the investigation of structural brain connectivity and tract integrity. Tracts have been recognized as crucial components in seizure generation and propagation (Berg and Scheffer, 2011, Richardson, 2012). Consequently, tractography may provide insight into different patterns of brain damage related to the laterality, type of epilepsy or epileptogenic lesion while along-the-tract analysis of diffusion metrics may also allow extraction of detailed information regarding neuropathology in epilepsy. It may have the potential to identify certain abnormal brain regions in individual patients (Martin et al., 2015), allow inferences about seizure onset zones or may even predict surgical outcomes (Keller et al., 2016).

### 4.2 Strengths and limitations

Our results closely correspond to those of time-consuming manual tractography studies (Ahmadi et al., 2009, Yogarajah et al., 2008, Rodrigo et al., 2007, Diehl et al., 2008, Lin et al., 2008). The automated tractography approach utilized here has several advantages over manual approaches, including an improved reproducibility of measurements, increased time efficiency and no reliance on trained human anatomists. The utilization of automated image analysis techniques, particularly those that are performed in native space, is favorable over manual ones if such techniques are incorporated into clinical evaluation of patients, particularly given the high levels of reproducibility. TRACULA uses reproducible tracking protocols validated on a set of healthy training subjects and has been shown to be sensitive to white matter changes in patients (Yendiki et al., 2011). We maintain that automated techniques for tract reconstruction will have important implications for studies aiming to characterize side of seizure onset and develop individual diagnostics in a clinical setting (Martin et al., 2015) due to its low demands on time and higher reliability (Leergaard et al., 2012). TRACULA constrains tractography to white matter voxels using an anatomically correct T1-w based segmentation mask. Diffusion weighted imaging may suffer from geometric distortions that can affect the accuracy of tract anatomy (Yendiki et al., 2011). The application of a b0/T1-w co-registration approach can mitigate the effects of potentially distorted voxels so that tracts can be successfully reconstructed. Furthermore, we assessed SNR and confirmed that mean SNR values for diffusion-weighted images were comparable across groups. However, motion was different across patients/controls and we therefore entered the TMI for all participants as a nuisance regressor into the analysis to mitigate confounding effects of motion differences between groups (Yendiki et al., 2013). Ultimately motion artifacts remain an important challenge for any DTI study, since macroscopic head motion can easily influence the microscopic diffusion measurements which DTI studies aim to characterize. As DTI-derived scalar metrics are dependent on the SNR measures of the data (Farrell et al., 2007) and motion (Yendiki et al., 2013), this has implications for studies investigating subtle differences between different types of disorders or when comparing patients to controls. Future tractographic studies should aim to assess image quality, possible motion confounds and employ appropriate statistics so that subtle differences in diffusion metrics between individual patients, groups of patients and controls can be confidently identified and accurate diagnostic markers can be developed. We show that TRACULA does not only provide detailed tractographic analysis strategies but also the possibility of detailed quality assurance and control giving researchers and clinicians a ready-to-use and sensitive tool for automated tractography. We exercise caution in attributing correlations between tract measurements and clinical data, in particular, duration of epilepsy, to seizure-induced brain damage given the cross-sectional nature of our study. Longitudinal studies are required to characterize the multifactorial relationship between seizures, clinical features of the disorder, medication, co-morbidities and white matter alterations. Longitudinal studies may provide insights into the etiology of diffusion alterations in temporal lobe white matter tracts.

### 4.3 Conclusion

The development and validation of automated tractography approaches is an important endeavour in neuroimaging research. We show that the automated tractography tool TRACULA is able to detect diffusion alterations in patients with TLE and may be used to investigate along-the-tract alterations in patients relative to controls. A tool which can resolve subtle intra-tract microstructural changes and relationships with morphometric abnormalities will likely afford important clinical information for individual patients. Our results suggest that white matter tract diffusion alterations are influenced by the chronicity and burden of the disorder, but not the extent of damage to the epileptogenic hippocampus. This may indicate that the development of hippocampal sclerosis and white matter tract alterations are dependent on different pathological developmental mechanisms.
